# miRNA profiling as a complementary diagnostic tool for amyotrophic lateral sclerosis

**DOI:** 10.1038/s41598-023-40879-y

**Published:** 2023-08-23

**Authors:** Jack Cheng, Wen-Kuang Ho, Bor-Tsang Wu, Hsin-Ping Liu, Wei-Yong Lin

**Affiliations:** 1 Graduate Institute of Integrated Medicine, College of Chinese Medicine, China Medical University, Taichung, 40402 Taiwan; 2 Department of Medical Research, China Medical University Hospital, Taichung, 40447 Taiwan; 3 Department of Senior Citizen Service Management, National Taichung University of Science and Technology, Taichung City, 40343 Taiwan; 4 Graduate Institute of Acupuncture Science, College of Chinese Medicine, China Medical University, Taichung, 40402 Taiwan

**Keywords:** Computational biology and bioinformatics, Amyotrophic lateral sclerosis

## Abstract

Amyotrophic lateral sclerosis (ALS), the most prevalent motor neuron disease characterized by its complex genetic structure, lacks a single diagnostic test capable of providing a conclusive diagnosis. In order to demonstrate the potential for genetic diagnosis and shed light on the pathogenic role of miRNAs in ALS, we developed an ALS diagnostic rule by training the model using 80% of a miRNA profiling dataset consisting of 253 ALS samples and 103 control samples. Subsequently, we validated the diagnostic rule using the remaining 20% of unseen samples. The diagnostic rule we developed includes miR-205-5p, miR-206, miR-376a-5p, miR-412-5p, miR-3927-3p, miR-4701-3p, miR-6763-5p, and miR-6801-3p. Remarkably, the rule achieved an 82% true positive rate and a 73% true negative rate when predicting the unseen samples. Furthermore, the identified miRNAs target 21 genes in the PI3K-Akt pathway and 27 genes in the ALS pathway, including notable genes such as BCL2, NEFH, and OPTN. We propose that miRNA profiling may serve as a complementary diagnostic tool to supplement the clinical presentation and aid in the early recognition of ALS.

## Introduction

ALS is a neurodegenerative disease that affects the central nervous system, characterized by motor neuron deficit and short life expectancy, but ALS can be challenging to diagnose, particularly in its early stages. Due to its rarity, physicians often consider more common illnesses before considering ALS, which can delay its diagnosis^1^. To improve early recognition, ALS diagnosis criteria have been proposed^2^ and continuously revised^3^. However, even the recently proposed Gold Coast criteria^4^ is still primarily based on clinical presentation, despite the fact that the genetic structure and biomarkers are gradually revealed by recent studies^5^. Additionally, newly developed predictive models, scales, and scoring systems can help patients and their physicians better understand the disease course. Although mechanism-based and potentially disease-modifying therapies are currently under clinical trials^5^, developing new diagnostic criteria and identifying genetic risk factors could also speed up the diagnostic process, and understanding the multisystem nature of ALS, including cognitive dysfunction and behavioral changes, is crucial for providing proper caregiving support and making end-of-life decisions.

ALS is currently divided into familial and sporadic categories. Familial ALS makes up 10–15% of cases and is inherited from family members with ALS or related syndromes like frontotemporal dementia^6^. About 70% of familial cases have mutations in known ALS genes. On the other hand, sporadic ALS makes up approximately 85% of cases and develops in patients without any family history of ALS. However, around 15% of sporadic ALS cases have private pathogenic mutations in known ALS genes, meaning they do not have a family history of ALS^6^. The cause of the remaining 85% of sporadic cases is unknown. Over 40 genes linked to ALS have been discovered, each varying in penetrance, frequency, and mode of inheritance. Among these genes, C9orf72, TARDBP, SOD1, and FUS are the most prevalent and have the highest penetrance^7^. Either toxic gain-of-function or loss-of-function mutations in these known genes are associated with ALS pathological processes. These mutations lead to protein aggregates forming, a key pathological feature in both sporadic and familial ALS cases^8^. The underlying pathophysiological processes can be broadly categorized into four main types: impaired RNA metabolism, altered proteostasis or autophagy, cytoskeletal or trafficking defects, and mitochondrial dysfunction^9^. For example, RNA metabolism is often affected by ALS-associated genes such as C9orf72, TARDBP, and FUS. Inclusions of TDP-43 and FUS can impair the normal function of DNA and RNA binding proteins, leading to significant changes in transcription and RNA processing. ALS-associated genes such as TDP-43 can also cause dysregulation of proteostasis and autophagy by preventing damaged protein clearance. Additionally, cytoskeletal and tubulin defects induced by mutant ALS genes like TUBA4A and PFN1 can interfere with axonal trafficking. Finally, SOD1 is a key gene linked to ALS and has been shown to cause mitochondrial dysfunction and increase oxidative stress, which is central to the development of the disease. Despite significant advances, our understanding of the complete molecular basis for the pathophysiology remains incomplete^5^.

MiRNAs are small non-coding molecules that post-transcriptionally regulate various biological processes, including neuronal function, development, and progression of diseases^10^. The expression level of miRNAs, including miR-1, miR-9, miR-133a, miR-133a/b, miR-142-3p, miR-149, miR-206, miR-223-3p, miR-326, miR-338-3p, miR-374b-5p, miR-424, and miR-451, in the serum, muscle, tissue, and cerebrospinal fluid of individuals with ALS differs from that of healthy controls^11–17^. These changes in miRNA expression may target the peripheral nerves, CNS, or skeletal muscles, potentially contributing to the etiology of ALS and playing a crucial role in its progression^18^. Therefore, Emde, et al., have proposed that different subtypes of ALS may share a common feature: the global dysregulation of miRNAs^19^. Thus, detecting changes in miRNA expression profile may serve as a valuable diagnostic biomarker to identify disease onset and progression. Additionally, identifying dysregulated miRNAs could offer a promising avenue for developing therapeutic approaches to treat ALS. However, due to ALS's polygenic nature and complexity, no single miRNA has been found universally dysregulated among previous studies. This fact makes it challenging to use miRNA as a diagnostic tool for ALS.

Here, we demonstrate the idea that ALS diagnosis could be assisted with the combination of the expression level of several circulating miRNAs. We established the judgement rule by machine learning using publically available dataset contributed by Magen and colleagues^20^, and showed that this rule model predicts 82% true positive (ALS) and 73% true negative (healthy control) in the blind dataset, and identified several novel miRNAs that target ALS genes.

## Methods

### Data source and batch correction

The raw counts of miRNA were downloaded from the National Center for Biotechnology Information (NCBI) Gene Expression Omnibus (GEO) database^21^ of the accession number GSE168714, contributed by Magen and colleagues^20^. The dataset was accessed at https://www.ncbi.nlm.nih.gov/geo/query/acc.cgi?acc=GSE168714 from March 1st to 3rd, 2023. The dataset comprises the annotated counts by small RNA-seq of the RNA extracted from the plasma of 253 ALS and 103 control^20^. The batch number of data collection and ALS/control state were also available with the raw count. To account for the differences in our study design compared to the original work of Magen and colleagues, we performed our own batch correction using the following method. Firstly, we temporarily excluded highly present miRNAs whose raw count represented more than 2% of the total counts (13 miRNAs excluded, as detailed in Supplementary File 1) during the calculation of batch correction coefficient. Secondly, we calculated the sum of miRNA counts for each sample. Thirdly, we determined the average sum of miRNA counts for each batch. We then defined a *batch coefficient* as the quotient of the *maximum average sum of all batches* divided by the *average sum of the batch*. Finally, we calculated the corrected count, including the temporarily excluded highly present miRNAs, by multiplying the raw counts by the *batch coefficient*.

### Machine learning

The strategy to identify the key miRNAs was adopted from our previous studies^22,23^. Briefly, the transposition of the corrected miRNA counts served as the input file to the machine learning program RapidMiner^24^. For clarity, bold italicized text is used to denote the terminology in RapidMiner. In the RapidMiner process setting, patient ID was used as *ID*; patient ALS state was used as *Label*, and the corrected miRNA counts were used as *Attributes* to perform training. *Rule Induction* was adopted as the algorithm, and the overall training and validation program was shown in Supplementary File 2, with the following parameters. The *Split Data* operator separated the sample library into 80/20 sets by shuffled sampling for model building and independent validation, respectively. *Rule Induction* was performed with the criterion of *information gain*, *sample ratio* of 0.9, *pureness* of 0.9, and *minimal prune benefit* of 0.25, while ten times of *Cross Validation* was used to improve the model. Finally, the *Apply model* operator utilized the 20% blind set to validate the generated *Rule Model*, and the *Performance* operator demonstrated the performance of the model.

### mRNA targets of miRNAs

DIANA-TarBase v7 was used to find the experimentally validated mRNA targets of miRNAs^25^. Data were accessed at http://diana.imis.athena-innovation.gr/DianaTools/index.php?r=tarbase/index from April 13th to 20th, 2023.

### Interaction network and enrichment

String-db version 11.5 was used to generate the interaction network and pathway enrichment^26^. Data were accessed at https://string-db.org/ from April 20th to 23rd, 2023. Interaction network was generated with highest confidence (0.9) and disconnected nodes in the network were hidden.

### Intersection analysis

Venny 2.0 was used to generate the Venn diagram^27^. Data was accessed at https://bioinfogp.cnb.csic.es/tools/venny/index2.0.2.html from April 20th to 23rd, 2023.

### Statistics

Student’s T-test was used to estimate the significance of the difference between two groups.

## Results

**Figure 1:**
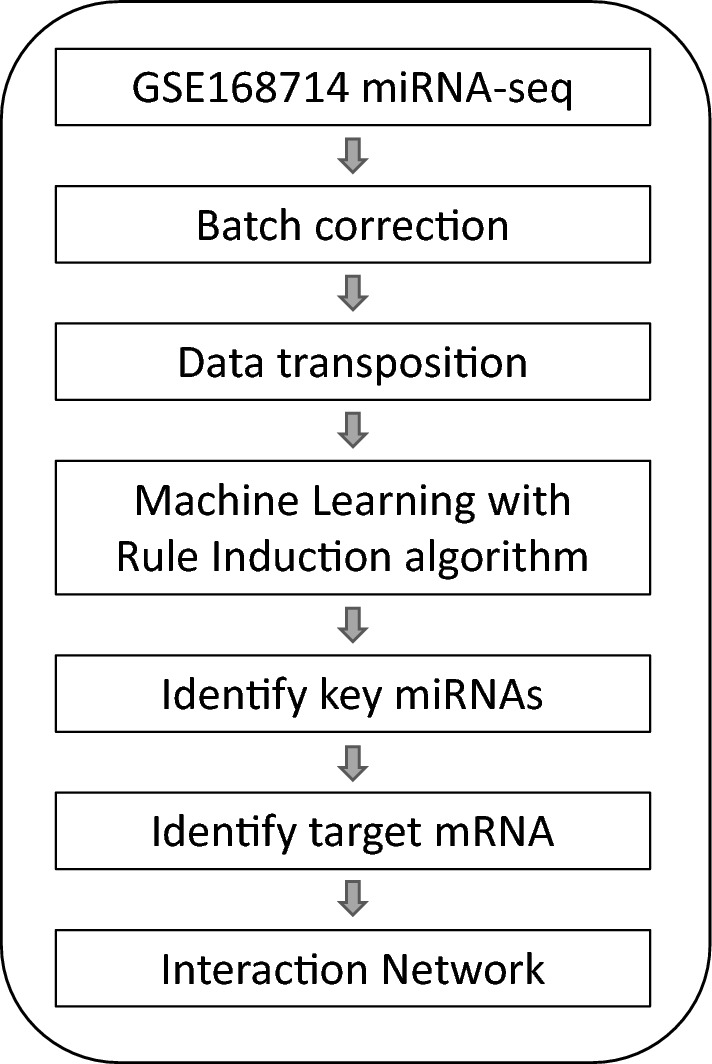
The workflow of this study.

**Figure 2:**
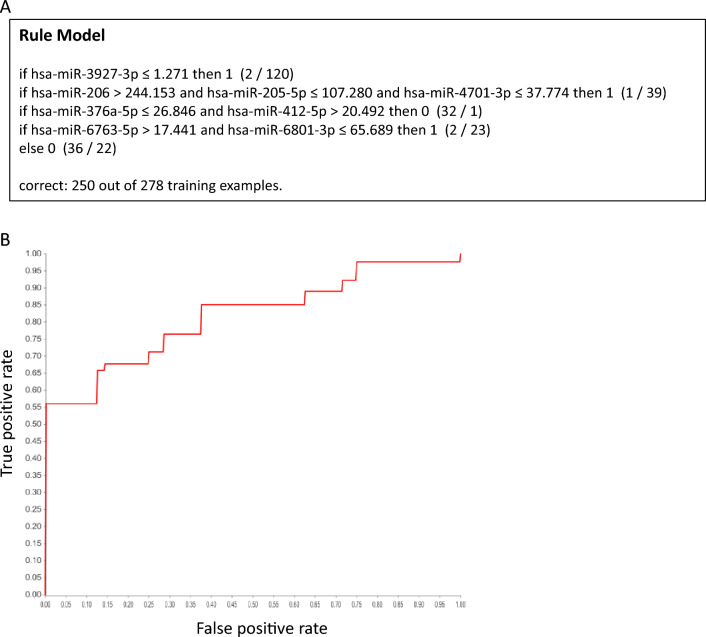
The predictive model generated by machine learning and its performance. (**A**) The rule model judging where a sample is ALS or control, where “1” denotes ALS and “0” denotes control. The value in the conditional expression is the batch-corrected count of the miRNA. (**B**) The ROC curve of the rule model, with AUC of 0.831.

**Figure 3:**
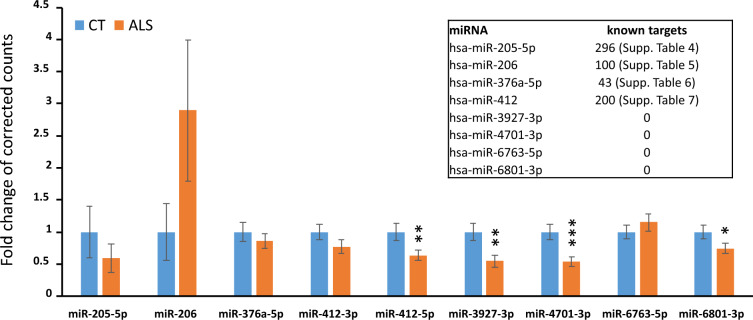
The miRNA expression fold change of the miRNAs identified by the rule model. Error bars stand for the standard error of the mean (SEM); while *, **, and *** stand for p-value < 0.05, 0.01, and 0.001 of Student’s T-test, respectively. The upper right panel shows the number of experimentally validated mRNA targets of miRNA, with the full list provided in the corresponding supplementary files.

Machine learning has been proven to be a powerful tool to identify genetic biomarkers in neurodegenerative disorders with complex and heterogeneous genetic factors, such as Alzheimer’s disease^22^ and Huntington’s disease^23^. Since ALS is also known for its complexity and heterogeneity genetic architecture, we applied machine learning on an ALS-control miRNA-seq dataset^20^ to establish a judgment rule compromising miRNA profiles to identify ALS biomarkers following the strategy illustrated in Fig. 1. After batch correction and transposition, batch-corrected counts of miRNAs of 253 ALS and 103 control was generated (Supplementary File 3), and served as the input file for machine learning, where 80% samples served as the training set, and 20% as the unseen testing set. The generated model is shown in Fig. 2A, compromising miR-205-5p, miR-206, miR-376a-5p, miR-412-5p, miR-3927-3p, miR-4701-3p, miR-6763-5p, and miR-6801-3p, and its ROC curve in the training stage is shown in Fig. 2B. This rule model predicts 82% true positive and 73% true negative in the unseen dataset. The expression fold change of the identified miRNAs is shown in Fig. 3, with miR-412-5p, miR-3927-3p, miR-4701-3p, and miR-6801-3p significantly down-regulated in ALS, but not the other four miRNAs. This reflects the idea that a major advance of machine learning over traditional comparative methods in identifying biomarkers from expression profiles^22^.

**Figure 4:**
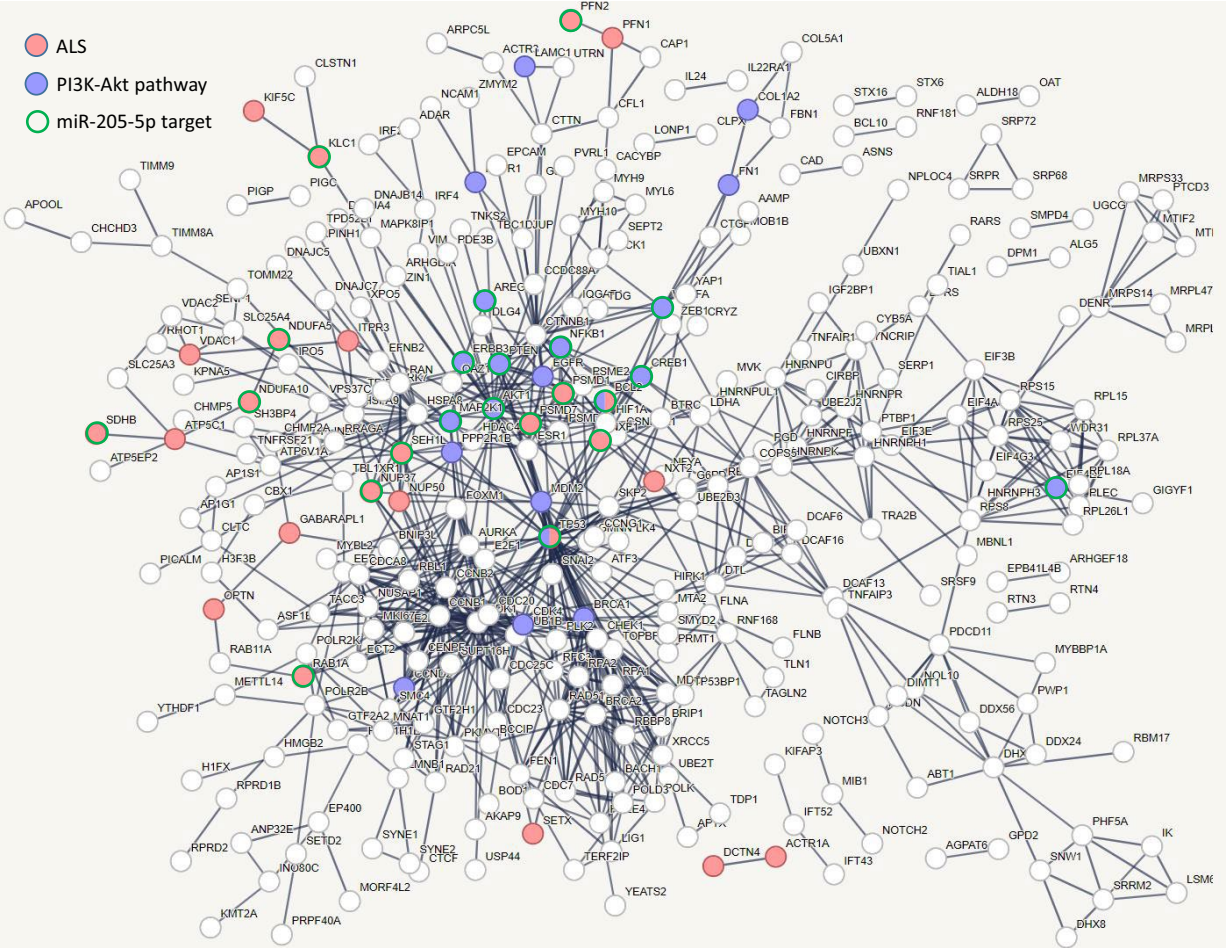
The protein interaction network of the mRNA targets of the identified miRNAs. Red balls indicate proteins in the Amyotrophic lateral sclerosis of KEGG, while blue balls indicate proteins in the PI3K-Akt signaling pathway of KEGG.

**Figure 5:**
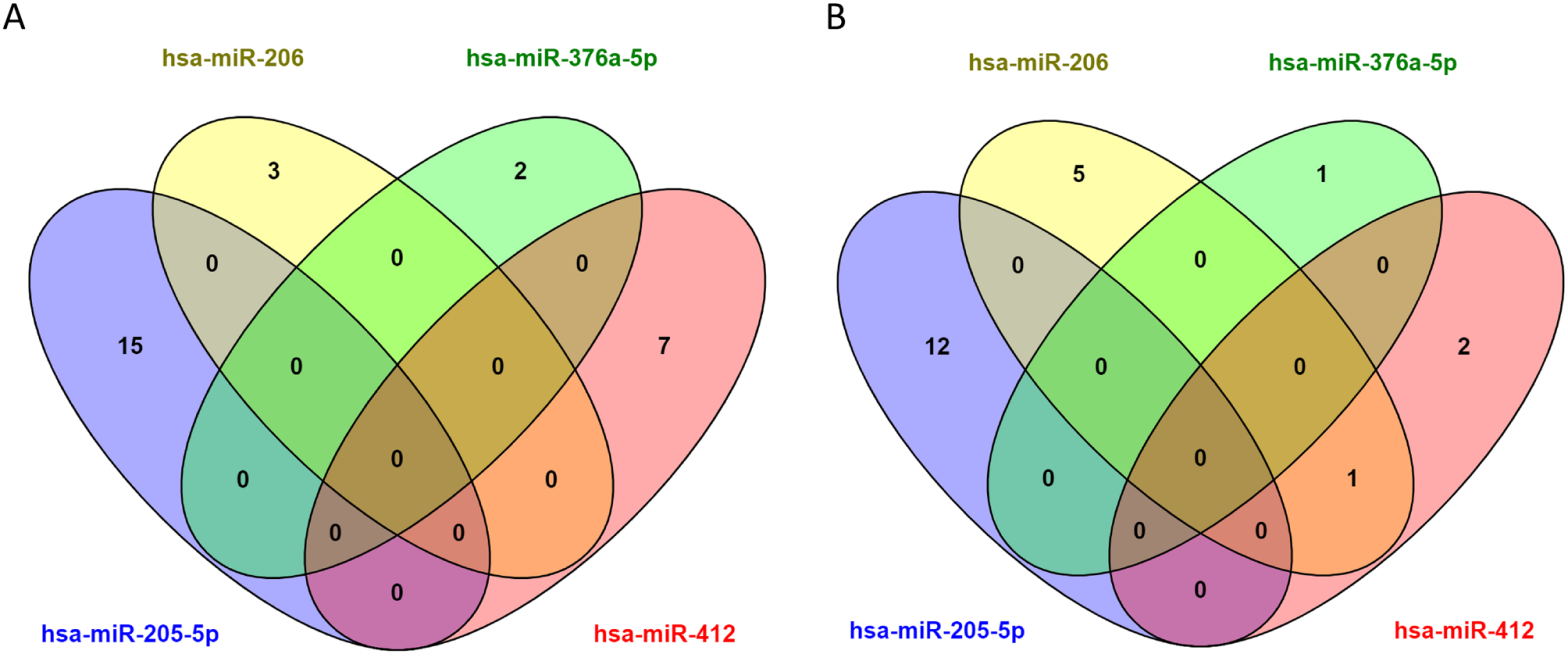
The Venn diagram showing that the miRNA-target sets are nearly mutually exclusive. (**A**) The ALS genes. (**B**) The PI3K-Akt genes.

To understand the role of miRNA-mediated biological processes in ALS pathology, we inquired the mRNA targets of miR-205-5p, miR-206, miR-376a-5p, miR-412-5p, miR-3927-3p, miR-4701-3p, miR-6763-5p, and miR-6801-3p in TarBase^25^, a curated database of miRNA-target interactions experimental evidence. Four miRNAs, i.e., miR-3927-3p, miR-4701-3p, miR-6763-5p, and miR-6801-3p, have no experiment-validated targets, while miR-205-5p, miR-206, miR-376a-5p, miR-412 have 296, 100, 43, and 200 targets, respectively (Supplementary File 4–7). Next, we showed that miRNA target genes enrich ALS and PI3K-Akt signaling pathways (Fig. 4, Supplementary File 8, 9), forming an interaction network with miR-205-5p targets occupying central hubs. Interestingly, The Venn diagram shows that the miRNA-target sets are nearly mutually exclusive in both ALS (Fig. 5A) and PI3K-Akt signaling pathway (Fig. 5B).

## Discussion

In this study, we proposed a diagnostic rule of ALS by the expression of miRNAs, including miR-205-5p, miR-206, miR-376a-5p, miR-412-5p, miR-3927-3p, miR-4701-3p, miR-6763-5p, and miR-6801-3p, where miR-206 has been reported in several previous ALS studies, while other miRNAs are novel in the ALS research field. Furthermore, these miRNAs mutually exclusively target genes in the ALS or PI3K-Akt pathways, which supports the idea that the combination of miRNAs, but not any single miRNA, may serve as a tool to facilitate ALS diagnosis. In the context of ALS’s heterogeneous genetics, we discuss the identified miRNAs’ and their target genes’ relevance to ALS below.

### Heterogeneity of ALS genetics

Recent advancements have greatly enhanced our comprehension of the genetic origins of familial ALS. Approximately 40–55% of familial ALS cases can be accounted for by variations in well-known ALS-linked genes^7^. While more than 50 potential causative or disease-modifying genes have been identified, pathogenic variants in SOD1, C9ORF72, FUS, and TARDBP are most frequent, whereas variants in other genes are relatively uncommon^28^. However, in cases of sporadic ALS, diagnostic progress has only elucidated a fraction of the cases, with the etiology remaining unexplained in over 90% of patients^29^. It is widely accepted that genetic risk factors play a significant role in sporadic ALS, with heritability estimated at approximately 60% based on twin studies^30^. However, despite extensive genetic association studies, identifying heritable genetic risk factors in sporadic ALS remains elusive.

Despite decades of research, ALS's underlying causative pathogenic mechanisms remain uncertain, particularly in sporadic cases. The development and progression of the disease are likely influenced by multiple factors rather than a single initiating event^31^. Moreover, genetic and phenotypic variations among patients pose challenges in comprehending and drawing conclusions about the general pathogenic mechanisms of ALS. Given the extensive number of genes and cellular processes implicated in ALS, numerous disease mechanisms have been proposed, including disruptions in RNA metabolism^32^, compromised protein homeostasis^33^, defects in nucleocytoplasmic transport^34^, impaired DNA repair^35^, mitochondrial dysfunction^36^, oxidative stress^37^, disturbances in axonal transport^38^, and oligodendrocyte dysfunction^39^. Further clarification is required to determine the timing and extent to which each of these mechanisms contributes to the pathogenesis of ALS.

As an attempt to see whether ALS clinical phenotypes could be differentiated by miRNAs, we constructed new cohorts of ALS patients from the same GSE168714 dataset by their clinical phenotypes: bulbar-onset or non-bulbar-onset, with 83 or 170 patients, respectively (Supplementary File 10), and these new cohorts of patients were subjected to de novo analysis in machine learning. However, the performance of the newly-established model was poor, with a recall rate of 75.9% and 24.6% for the cohorts, respectively. Thus, machine learning could not differentiate the cohorts of ALS, at least under the present condition. We further analyzed whether the identified miRNAs express differentially in the cohorts of ALS (Supplementary File 11). We found that miR-206 and miR-205 express differentially in the cohorts of ALS, but not significantly. We suspect that the sample size is the bottleneck to uncover the nature of the ALS cohorts. We also summarized other publicly-available miRNA datasets (Supplementary File 12). However, their sample size is insufficient for an independent machine learning study, and none of the datasets meets the requirement for validation of the current model that all eight miRNAs used in the machine learning model must be available.

### miR-206

miR-206 participates in various stages of muscle differentiation, encompassing alternative splicing, DNA synthesis, and cell apoptosis^40^. During development, miR-206 hinders the activity of Pax7 and Pax3, effectively restricting the proliferative potential of satellite cells while promoting their differentiation into myogenic progenitor cells. Conversely, reducing miR-206 leads to the overexpression of Pax7 and Pax3, which consequently inhibits the differentiation of myoblasts. As Pax7 and Pax3 are known pro-survival factors, the down-regulation of miR-206 can induce apoptosis^41^. Thus, miR-206 plays a protective role and facilitates the regeneration of neuromuscular junctions following acute nerve injury, particularly in the context of ALS^42^. Knock-out of miR-206 delays and mutilates muscle reinnervation in ALS mouse models of SOD mutant^43^. Recent findings indicate elevated levels of miR-206 in the plasma of ALS patients and could indicate of disease progression^44,45^.

### miR-205

miR-205 exhibits significant expression levels in various human epithelial tissues, including the breast, prostate, skin, eye, and thymus. Its primary role in these tissues is crucial to tissue morphogenesis and homeostasis. Specifically, it upholds the epithelial phenotype by directly targeting two transcription factors: zinc finger E-box-binding homeobox 1 (ZEB1) and ZEB2, which repress E-cadherin and other genes associated with polarity^46^. During the early stages of embryonic development, miR-205 is expressed in trophoblasts, where it regulates placental development by suppressing the Mediator of RNA polymerase II transcription subunit 1 (MED1)^47^. Moreover, in embryonic development, miR-205 governs the differentiation of extraembryonic endoderm and spermatogenesis by influencing cell migration and adhesion genes^48^. In the mammary gland, miR-205 displays high expression levels in the basal stem cells. Overexpression of miR-205 has been shown to induce the expansion of the progenitor cell population while reducing cell size and promoting cellular proliferation. These effects are achieved by repressing PTEN^49^. In this context, miR-205 regulates the production of the basement membrane protein complex laminin-332 and its receptor integrin-β4, thereby ensuring proper tissue polarity and morphogenesis^50^. In the skin epidermis and stratified epithelia of the esophagus and tongue, miR-205 has been found to play a significant role in expanding the stem cell population through its regulation of PI3K signaling^51^. Additionally, by influencing the same signaling pathways, miR-205 enhances the migration of human epidermal and corneal epithelial keratinocytes, thereby contributing to wound healing and corneal development^52^.

### miR-376a and miR-412

The physiological roles of miR-376a and miR-412 are not fully understood yet, despite some studies reported its participation in cancer and neurological disorders. For example, increased levels of miR-376a have been observed in the T cells of patients with multiple sclerosis (MS)^53^, and miR-412 may inhibit clear cell renal cell carcinoma progression^54^. Conversely, in the late-onset form of Alzheimer's disease (LOAD), miR-376a has been identified as down-regulated in the brain^55^. Meanwhile, the expression change of miR-412 has been mentioned in the brain of alcohol use disorder^56^, and also in Alzheimer's disease^57^.

### BCL2

BCL2 is targeted by miR-205^58^ and controls caspase activation and the initiation of programmed cell death^59^ and thus regulates neuronal development and neurodegeneration^60^. Several lines of evidence show that BCL2 probably involves in ALS pathological progression. Epidemiologic studies found altered expression of BCL2 in ALS spinal cord motor neurons^61^ and post‐central gyrus^62^. In vivo studies showed that overexpression of BCL2 prolongs the survival of the ALS mouse model^63^ and improves neuromuscular function^64^. In vitro studies revealed that ALS-associated mutant SOD1 aggregates BCL2^65^ and advocates BCL2 conformational changes^66^.

### NEFH

Neurofilament heavy polypeptide (NEFH) is one of the three intermediate filament proteins forming neurofilaments^67^. NEFH is targeted by miR-205^68^ and could be phosphorylated by GSK3β^69^ and regulate the Akt-β-catenin pathway^70^. Moreover, epidemiological study showed that NEFH mutation^71,72^ and expression^73^ is associated with ALS. Besides, NEFH mutation or expression is associated with other disorders of central or peripheral neural system, including schizophrenia^74^, alcoholics^75^, and Charcot-Marie-Tooth neuropathy^76^.

### OPTN

OPTN, also known as optineurin, is a highly conserved protein in various species^77^. OPTN is target by miR-205^68^ as well. It plays diverse roles in vesicular trafficking, NFKB/NF-κB signaling, and autophagy. Specifically, OPTN has been identified as an autophagy receptor that facilitates the connection between ubiquitinated autophagy substrates and MAP1LC3/LC3-positive phagophore membranes^78^. Furthermore, mounting evidence suggests that OPTN acts as an inducer of autophagy, initiating the autophagic process^79,80^. Moreover, studies indicate that OPTN's involvement in autophagic initiation can commence as early as the formation of autophagosomal membranes^81,82^. These groundbreaking findings underscore the multifunctional role of OPTN as a potential autophagy receptor throughout the autophagic process, expanding beyond its traditional perception as a receptor operating solely at a single stage of autophagy. OPTN is gathering attention in ALS research, since variants of this gene are associated with ALS^83–85^. Moreover, OPTN mutant induces neuronal cell death by mediating mitophagy^86^, autophagy and ER stress^87^. Interestingly, OPTN mutation might be the common cause of ALS and corticobasal syndrome (CBS)^88^.

### PI3K in ALS

The PI3K-askt signaling pathway governs metabolism, cell survival, motility, transcription, and cell-cycle progression. In recent years, studies have revealed the involvement of the PI3K-Akt signaling pathway in neurodegenerative diseases. For instance, butylphthalide has been shown to activate the PI3K-Akt/GSK-3β signaling pathway in an ischemic cerebral infarction model, reducing nerve function damage and protecting local nerve cells^89^. Therefore, therapeutic strategies for ALS targeting the PI3K-Akt pathway has been shown to increase anti-apoptotic protein expression levels, reduce pro-apoptotic protein expression levels, and improve cell survival rate and mitochondrial function in ALS^90^. Moreover, studies by Xiang and colleagues have found that AEG-1 can regulate the PI3K-Akt pathway^91^, and the absence of AEG-1 in ALS motor neurons inhibits the PI3K-Akt pathway and increases cell apoptosis^91^. Thus, dysregulated miRNAs may promote ALS pathology by mediating PI3K-Akt signaling pathway.

## Conclusion

In sum, we showed that a set of miRNA expressions could serve as a diagnostic tool for ALS, and these miRNAs target ALS and PI3K-Akt pathways in a mutually exclusive way. The key miRNAs include miR-205-5p, miR-206, and miR-376a-5p, while key targets are BCL2, NEFH, and OPTN. We propose that miRNA profiling may facilitate clinical presentation in the early recognition of ALS.

### Supplementary Information

Supplementary Information.

## Data Availability

All data in this study are included in the supplementary data. The raw data used for machine learning is shown in Supplementary File 3, and the first two rows (file descriptions) must be removed before use. The raw counts of miRNA are also available from the National Center for Biotechnology Information (NCBI) Gene Expression Omnibus (GEO) database of the accession number GSE168714.

## Code availability

The machine learning platform RapidMiner Studio is available at https://rapidminer.com/. The process is shown in Supplementary File 2, and the parameters are in the “Methods” section.
